# Risk factors associated with pulmonary tuberculosis relapses in Cali, Colombia

**DOI:** 10.7705/biomedica.5061

**Published:** 2020-08-20

**Authors:** Cindy Córdoba, Paola A Buriticá, Robinson Pacheco, Anyela Mancilla, Augusto Valderrama-Aguirre, Gustavo Bergonzoli

**Affiliations:** 1 Facultad de Ciencias de la Salud, Grupo de Investigación en Epidemiología y Servicios, Programa de Maestría en Epidemiología, Universidad Libre - seccional Cali, Cali, Colombia Universidad Libre Facultad de Ciencias de la Salud Grupo de Investigación en Epidemiología y Servicios Universidad Libre Cali Colombia; 2 Instituto de Investigaciones Biomédicas, Cali, Colombia Instituto de Investigaciones Biomédicas Cali Colombia; 3 Fundación para la Producción y Gestión del Conocimiento, Cali, Colombia Fundación para la Producción y Gestión del Conocimiento Cali Colombia; 4 Universidad Santiago de Cali, Cali, Colombia Universidad Santiago de Cali Universidad Santiago de Cali Cali Colombia

**Keywords:** Pulmonary tuberculosis, recurrence, body mass index, Colombia, tuberculosis pulmonar, recurrencia, índice de masa corporal, Colombia

## Abstract

**Introduction:**

Relapses in tuberculosis occur due to endogenous reactivations or exogenous reinfections and represent up to 27% of tuberculosis cases. Its importance lies in the risk of the appearance of multidrug-resistant *Mycobacterium tuberculosis* strains. According to the reports published in 2011 by the Colombian *Instituto Nacional de Salud*, there were 572 relapse cases reported in the country, i.e., a rate of 4.9%. Data of the tuberculosis control program from the *Secretaría de Salud Municipal* in Cali reported a relapse rate of 6%, higher than the national one, during 2013 and 2014.

**Objective:**

To determine the risk factors associated with relapse in patients with pulmonary tuberculosis in Cali.

**Materials and methods:**

We conducted an observational, analytical, and case-control study (1:1), which comprised 81 cases of pulmonary tuberculosis relapses detected in 2013 and 2014. Additionally, we collected data on socio-demographic and clinical variables, as well as lifestyle and health services, to identify the potential risk factors associated with tuberculosis relapses. We used logistic regression to identify the risk factors.

**Results:**

After adjustments for some variables, our multivariate logistic regression analysis showed that the body mass index (BMI) (OR=0.90, 95%CI: 0.81-0.99) and population density (OR=0.99, 95%CI: 0.98-1.00) were inversely associated with tuberculosis relapses. Alcohol consumption increased the likelihood of tuberculosis relapse (OR=5.56, 95%CI: 1.18-26.26).

**Conclusions:**

Body mass index and population density were inversely associated with pulmonary tuberculosis relapses in Cali. On the contrary, alcohol consumption increased the likelihood of tuberculosis relapses.

## Resumen

**Introducción.:**

Las recaídas en la tuberculosis se deben a reactivaciones endógenas o reinfecciones exógenas y alcanzan hasta el 27 % de los casos. Su importancia radica en el riesgo de aparición de cepas de *Mycobacterium tuberculosis* resistentes a múltiples fármacos. Según informes del Instituto Nacional de Salud de Colombia, en el 2011 se reportaron 572 recaídas, lo que representa un porcentaje del 4,9 %. Datos del programa de tuberculosis de la Secretaría de Salud Municipal de Cali registraron una tasa de recaídas del 6 % durante el 2013 y el 2014, lo que supera la tasa nacional.

**Objetivo.:**

Determinar los factores asociados con la recaída en pacientes con tuberculosis pulmonar.

**Materiales y métodos.:**

Se hizo un estudio observacional, analítico y de casos y controles en pacientes diagnosticados con tuberculosis pulmonar detectados entre el 2013 y el 2014. Se estudiaron las variables sociodemográficas, clínicas, de estilo de vida y las relacionadas con el programa y los factores de riesgo; se determinaron utilizando regresión logística.

**Resultados.:**

Después de ajustar por otras variables, la regresión logística evidenció dos factores inversamente asociados con las recaídas: el índice de masa corporal (OR=0,90; IC95%: 0,81-0,99) y la densidad poblacional en las comunas (OR=0,99; IC95%:0,99-1,00. El consumo de alcohol aumentó la probabilidad de recaída (OR=5,56; IC95%: 1,18 - 26,26).

**Conclusiones.:**

El índice de masa corporal y la densidad poblacional se asociaron inversamente con las recaídas por tuberculosis pulmonar en Cali. El consumo de alcohol estuvo directamente relacionado.

Relapses in tuberculosis occur due to endogenous reactivations or exogenous reinfections with a prevalence rate of up to 27%. Their importance lies in the risk of the appearance of multidrug-resistant *Mycobacterium tuberculosis* strains. According to the reports published in 2011 by the Colombian *Instituto Nacional de Salud*, there were 572 relapse cases in the country, i.e., a rate of 4.9% ^1^. The data from the tuberculosis control program of the *Secretaría de Salud Municipal* in Cali reported a relapse rate of 6%, higher than the national one, during 2013 and 2014.

There are several and well-documented risk factors associated with pulmonary tuberculosis relapses all over the world. These factors include failure to adhere to treatment, the persistence of pulmonary cavitation, and multidrug resistance to medications ^2^. The importance of socio- demographic, cultural, clinical, and behavioral determinants is still under evaluation. Studies conducted in Colombia and other Latin American countries have shown that residing in urban areas, overcrowding, and irregular treatment are some of the factors associated with relapses in patients with pulmonary tuberculosis as confirmed also by bacilloscopy ^3^. Toledano, *et al.,* indicated that high relapse rates were associated with high population density ^4^ along with alcoholism and smoking ^5^. In 2015, a study found that malnutrition, intra-home tuberculosis infection, urban marginal people, harmful habits such as alcoholism and smoking, and presence of comorbidities including diabetes mellitus, chronic renal failure, and silicosis were related to tuberculosis relapses ^6^.

The nutritional status is another factor that needs careful consideration. Body mass index (BMI) has been used as a proxy indicator of nutritional status for examining its association with relapses in patients with pulmonary tuberculosis ^7^. A study conducted in India, in 2014, found that malnutrition increased the incidence of TB cases in men and women of the participant tribes ^8^.

Cali has a high tuberculosis burden and the relapse rate is higher than the national one. However, to date, there are no published studies that focused on identifying the factors associated with relapses. Consequently, there is a lack of information for informed decisions to control relapses. This situation implies a serious public health concern since there are treated patients who continue to transmit the infection in their respective communities and elsewhere. It also represents an additional cost for control programs as these patients must undergo additional procedures such as microbiological cultures, molecular tests, and antimicrobial sensitivity tests ^9^. In this context, the purpose of this study was to determine the risk factors associated with relapse in patients with pulmonary tuberculosis in Cali, Colombia.

## Materials and methods

We conducted an observational, analytical, and case-control study (1:1) in a sample of patients from the tuberculosis control program database between January, 2013, and December, 2014, among whom we detected 81 cases and selected 81 controls, a sample size with an 85% statistical power. The criteria used to estimate the power were P_o_=0.35, P_1_=0.57, OR=2.5 ^10^^,^^11^.

### Inclusion and exclusion criteria and case and control definitions

We included patients with a confirmed diagnosis of pulmonary tuberculosis by bacilloscopy residing in Cali with at least 80% of the variables under study recorded in the database. We excluded the records of patients who were registered in the control program and those who had left the program or had had a therapeutic failure.

The relapse cases were defined as those patients readmitted to the control program due to relapse as defined by the World Health Organization (WHO). A patient previously treated for tuberculosis was declared as cured based on three successive negative smears or negative cultures in the four months after treatment. Controls were defined as those with a first episode of tuberculosis diagnosed in the same year as the case and with no relapse after the same follow-up period ^12^. Study subjects were selected from the same database by random sampling ^13^^,^^14^.

### Risk factors

The independent variables collected were grouped as follows:

1) Socio-demographic factors: sex, age, self-reported ethnic group (indigenous, mestizo or afro-descendant), socio-economic status, marital status, level of education, job, vulnerability condition (street dweller or displaced), commune (an administrative subdivision of the city), overcrowding, and social security affiliation regime;

2) clinical and biological factors: BMI, chest radiological findings, coinfection with HIV, diabetes, and kidney disease;

3) lifestyle factors: tobacco use, alcohol consumption, and use of illicit drugs such as marijuana, heroin, and cocaine;

4) healthcare-associated factors: variables related to the intake of antiretroviral drugs, rotation of control program officials, and duration of the tuberculosis symptoms.

### Data management and analysis

We collected data from three different sources: the tuberculosis control program databases, the individual treatment card, and medical records. Then, we entered them into Microsoft Excel™ tables (version 2010, Redmond, WA) with double digitization to ensure the quality of data and, then, we exported them to the SPSS™, version 21 package ^15^.

We made an exploratory analysis of each of the variables to evaluate their distribution. Proportions were estimated with their respective 95% confidence interval values. In the case of quantitative variables, we used the measurements of central tendency and variability. The bivariate analysis aimed at evaluating numerical problems such as collinearity, complete separation, and cells with zeroes or less than five expected observations.

In this phase, we identified the variables that would be subjected to the multivariate analysis as those having a randomness effect on the association between the dependent or response variable and each of the independent or predictive variables.

To compare the characteristics of cases and controls we used either the Student or the chi-square test depending on the measurement scale used in each variable. We evaluated each test with a level of significance of 5% and their respective 95% confidence intervals.

A multivariate analysis was conducted using a binary logistic regression model to evaluate the goodness of fit of the global model and to identify the factors associated with pulmonary tuberculosis relapses. The stepwise procedure was used to select the independent variables by using an alpha value equal to 0.15 to enter the model and one equal to 0.10 to stay. Additionally, the statistically insignificant variables in the bivariate analysis reported by other authors as important biological or public health factors related to pulmonary tuberculosis relapses were also included in the statistical model.

Logistic regression is based on several assumptions. Given that the dependent variable is the proportion of individuals who present a binary attribute and such proportion follows a binomial distribution, most of the linear regression assumptions do not apply in logistic regression. Logistic regression assumes a linear regression between the logit of the dependent variable and all independent variables, which, in turn, can be binary, ordinal, and interval. The observations must be independent and there should be an absence of multicollinearity between independent variables and the outliers responsible for the leverage of estimates. Multicollinearity should be checked and explained. All these problems can be prevented through good study design and analysis.

The adequacy of the model was tested based on the analysis of deviance. This model validation was performed by computing the maximum likelihood of a null model and taking their ratio to the model we were interested in developing. Based on this ratio, a measure called deviance is defined, which is twice the difference between the likelihood of a perfectly fitted model and the likelihood of the model we are interested in developing. This quantity is known as the log-likelihood ratio because the difference between the two likelihoods is equal to the logarithm of their ratio. Thus, the better the model fits the data, the smaller the deviance and the log-likelihood ratio.

Therefore, all we must find is the difference between the minus 2 likelihood of the null model and the model we are interested in developing. This result is known as the log-likelihood ratio test, which follows a chi-square distribution with the degrees equal to the number of independent variables in the model under evaluation.

We also evaluated the effectiveness of the model to describe the outcome variable, a procedure known as goodness of fit. These measures are based on the difference between the observed and the fitted values. Therefore, the smaller the difference, the better the fit of the model. We used the Hosmer- Lemeshow test and through multiple modulations, we demonstrated that when J equals n and the fitted logistic regression model is the correct model, the distribution of this test is well approximated by the chi-square distribution with (g - 2) degrees of freedom ^14^^,^^16^^,^^17^.

After the statistical analysis, we interpreted and contextualize the results to respond to the research question on the risk factors associated with tuberculosis relapse and to identify practical public health interventions. The measurements of association were the OR and its respective 95% CI for each risk factor identified as associated with the relapses.

### Ethical issues

The research ethics committee of *Universidad Libre* in Cali reviewed and endorsed this research project. Health authorities in Cali provided written consent for using their database. Our study used records and, therefore, it is classified as free of risk in Article 11 of Resolution 8430 of 1993 issued by the *Ministerio de Salud* from Colombia.

Data access was coded to comply with the guidelines of the Helsinki Declaration on confidentiality and protection of participants’ identity and personal data.

## Results

### Comparison of socio-demographic factors

The mean age was close to 50 years in both groups and its distribution was almost equal both in cases and controls which guaranteed that this variable would not be confounding. Male and mestizo ethnic groups were predominant in both groups with 63.0% and 80.2%, respectively in cases, and 58% and 87.7%, respectively in controls. The majority of participants belonged to low socio-economic strata, with 66.7% for both groups, i.e., they belonged to the subsidized healthcare regime. The predominant educational levels were elementary and high school (39.2% and 47.2%, respectively for cases, and 35.1% and 40.2%, respectively for controls). Unemployment was 47.5% and 38.3% for cases and controls, respectively. Most of the participants were single (33.3% and 38.3% for cases and controls, respectively).

The incidence rates by commune were similar in both groups (60.7% and 60.8% for cases and controls, respectively). None of these variables yielded statistical significance except the population density in communes, which was much higher in the control group (table 1).

**Table 1:** Characteristics of the study subjects

Variable	Descriptor	Cases (n=81) (%)	Controls (n=81) (%)	p
Socio-demographic factors				
*Age	Mean	51.4	51.7	0.90
Sex	Male	63.0	58.0	
	Female	37.0	42.0	0.50
Ethnic group	Afro-Colombian	17.3	11.1	0.40
	Brown	80.2	87.7	
	Indigenous	2.5	1.2	
Socioeconomic status	High	2.5	1.2	1.00
	Medium	30.9	32.1	
	Low	66.7	66.7	
Civil status	Single	33.3	38.3	0.20
	Married	30.9	37.1	
	Free union	17.3	8.6	
	Divorced	4.9	8.6	
	Widower	13.6	7.4	
Education level	None	5.4	11.7	0.60
	Elementary school	39.2	35.1	
	High school	47.3	40.3	
	Technical	4.1	9.1	
	Academic	4.0	3.8	
Occupation	Employee	52.0	61.7	0.10
	Unemployed	47.0	38.3	
	Missing data	0.6	--	
Health regime	Contributory	23.5	38.3	0.10
	Subsidized	64.2	50.6	
	Linked	12.3	11.1	
TB incidence by commune	Mean	60.7	60.8	1.00
Population density by commune	Mean	283.8	525.9	0.03
Clinical-biological factors				
Weight	Mean	52.6	54.5	0.23
Height	Mean	1.64	1.63	0.48
BMI	Normal weight	49.0	48.0	0.53
	Subnormal weight	23.0	28.0	
Positive radiological findings	Positives	92.6	91.4	0.60
	Negatives	1.2	3.7	
	Missing data	6.2	4.9	
HIV coinfection	Yes	11.1	7.4	0.40
	No	85.2	91.4	
	Missing data	3.7	1.2	
Diabetes	Yes	8.6	3.7	0.10
	No	91.4	96.3	
Renal disease	Yes	1.2	0.0	0.30
	No	98.8	100	
Lifestyle factors				
Tobacco use	Yes	11.1	3.7	0.01
	No	88.9	96.3	
Alcohol consumption	Yes	12.3	2.5	0.01
	No	87.7	97.5	
Drug use	Yes	3.7	0.0	0.10
	No	96.3	100	
Health services factors				
Intake of antiretroviral drugs	Yes	7.4	7.4	0.20
	No	0.0	3.7	
	Missing data	92.6	88.9	
Rotation of health care personnel	Yes	23.5	21.0	0.70
	No	76.5	79.0	
Symptomatology elapsed	Mean	108.5	96.8	0.70

* Mean

### Comparison of clinical and biological factors

Weight was similar in both groups with mean values of 52.6 kg and 54.5 kg for cases and controls, respectively (Student’s t=- 1.20, DF=60, p=0.23). Height was also similar in the groups with mean values of 1.64 m and 1.63 m for cases and controls, respectively (Student’s t=0.70, DF=156, p=0.48). As expected, there were high percentages of positive radiological findings in both groups: 92.6% and 91.4% for cases and controls, respectively. The majority of participants had no coinfections with HIV, diabetes or kidney disease. There were no statistically significant differences among these variables (table 1).

### Comparison of lifestyle factors

Cases had a higher prevalence of smoking (11.1%) than controls (3.7%).

Alcohol consumption was higher in the cases (12.3%) than the controls (2.5%). The rates of illicit drug use were 3.7% and 0.0% in cases and controls, respectively. Nevertheless, the difference was only statistically significant for alcohol consumption (c^2^=5.76, DF=1, p=0.01) and population density (Student’s t=-2.99, DF=86.6, p=0.000); the equality of variances was not assumed for F-Snedecor=37.2, p=0.000.

### Comparison of health services-related factors

Although none of the variables turned out to be statistically significant, it is important to point out that lapses between the first symptoms and diagnosis were, on an average, 108.5 days and 96.8 days for cases and controls, respectively. This result was not statistically significant (Student’s t=0.43, DF=160, p=0.66) (table 1).

After the bivariate analysis, the following variables were included in the logistic model: population density (p=0.03), diabetes (p=0.19), tobacco consumption (p=0.07), alcohol use (p=0.02), drug use (p=0.08), HIV treatment (p=0.11), and malnutrition (p=0,97) (table 2).

**Table 2:** Associated risk factors

Variable	95% CI			
	OR	SE	Lower limit-Upper limit	
Alcohol consumption	5.56	0.792	1.18	26.26
Body mass index	0.90	0.054	0.81	0.99
Population density	0.99	0.001	0.98	1.00

SE: Standard error

The logistic regression results demonstrated that alcohol consumption (OR=5,56; 95%CI=1,18-26,26), BMI (OR=0,90; 0,81-0,99), and population density (OR=0,99; 0,98-1,00) were the three major risk factors associated with tuberculosis relapses in this population (table 3).

**Table 3:** Model summary

Test	Result
−2 Log likelihood	203,559a
Cox & Snell R Square	0.093
Nagelkerke R Square	0.124

a Estimation terminated at iteration number 5 because parameter estimates changed by less than 0.001.

The logistic model adjusted the data well. Moreover, the Hosmer-Lemeshow test yielded the following values: c^2^= 9.9, DF=8, p=0.29. The Omnibus test for the regression coefficients in the model showed statistical significance and yielded the following values: c^2^=15.5, DF=3, p=0.001. The findings demonstrated that at least one of the regression coefficients significantly contributed to tuberculosis relapse thereby increasing or decreasing the probability of its occurrence. Table 3 provides a summary of the logistic model.

## Discussion

Our findings show that BMI and population density were inversely related to the occurrence of pulmonary tuberculosis relapses in Cali, Colombia, after adjusting other factors. In fact, alcohol consumption was positively related to tuberculosis relapses.

Also, Muñoz, *et al.,* pointed out that a decrease in BMI led to an increased risk of tuberculosis. This relationship remained after controlling for socio- economic level, high alcohol consumption, history of gastrectomy, and prior vaccination with BCG ^7^. In our study, having a normal nutritional status, which was measured via BMI, acted as a protective factor against tuberculosis relapses.

In 2014, Bhargava, *et al*., estimated that 60% of the incidence of tuberculosis among women living in the central and eastern states of India was attributable to malnutrition ^8^. However, we did not obtain the same results.

In 2005, Thomas, *et al.,* conducted a prospective study to measure the relapse rate in patients who had completed the tuberculosis treatment and were cured in the context of the control program. They also aimed to identify the risk factors associated with relapses. They found that irregularity in the treatment (OR=2.5; 95%CI: 1.4-4.7), initial drug resistance profile (OR=4.8; 95%CI: 2.0-11.6), smoking (OR=3.1; 95%CI: 1.6-6.0), and alcoholism (OR=2.3; 95%CI: 1.3-4.1, p=0.01) ^18^ were all associated with tuberculosis relapses.

In 2015, San Martín, *et al*., reported that tuberculosis relapses were associated with malnutrition (OR=6.57), bacillary load (OR=4.03), intra- domiciliary tuberculosis infection (OR=24.75), urban marginal settings (OR=2,7), and harmful habits, such as alcoholism (OR=5.26) and smoking (OR=3.6) ^6^.

In 2010, Toledo, *et al.,* conducted a cross-sectional study and found that the most prevalent toxic habit in patients with tuberculosis was alcohol consumption (83.3%). Also, in their discussion section, they mentioned that the prevalence of patients with relapses might be related to a high population density ^4^, which is similar to our findings.

The factors associated with tuberculosis relapses in the abovementioned studies were classified as behavioral factors, which correspond to unhealthy lifestyles. This finding is consistent with the health determinants listed by the World Health Organization (WHO), which include stress, social exclusion, addictions such as alcohol consumption, drugs, and tobacco use, unhealthy diet, overcrowding, and other adverse life conditions like multidimensional poverty (measured by poor housing conditions), low income, and unemployment ^19^^,^^20^. These health determinants can be directly related to some of the socio-demographic factors found in our study; however, they did not show any statistical significance. Unemployment (47%), low socioeconomic status (66.7%), and low educational level (39.7%) showed a greater prevalence among the cases, which agrees with the report published by Millet in 2012 on factors affecting the epidemiology of tuberculosis, such as poverty, overcrowding, and malnutrition, along with social inequalities, HIV infection, and drugs or alcohol abuse ^20^.

Gadoev, *et al.,* found that age between 35 and 55 years, a positive smear for pulmonary tuberculosis, some comorbidities including chronic obstructive pulmonary disease were associated with tuberculosis recurrence ^21^.

Kim, *et al.,* found that relapses were associated with age, HIV infection, being a foreigner, and consumption of psychoactive substances ^22^.

On the contrary, Núñez, *et al.,* found that failures in tuberculosis treatment, living far away from a health center, and having a conflicting family environment increased the risk of tuberculosis relapses ^23^.

Mehdi, *et al.,* pointed out that some factors of the host that do not depend on tuberculosis control program activities: gender, malnutrition, diabetes, kidney failure, and HIV infections predispose to relapsing ^24^ while Rutledge, *et al.,* found that when not supervised, multidrug resistance and non- adherence to treatment were related to tuberculosis relapse ^25^.

Based on a systematic review, Naidoo indicated that the distinction between relapse and reinfection is of paramount importance in addressing the burden of recurrent tuberculosis. High rates of relapse demand renewed interventions to improve individual patient care whereas high rates of reinfection demand improved infection and epidemic control measures. Urgent attention is required to address the challenges of adherence, such as social and health care worker support systems and step-down management facilities ^26^.

The major disagreement between our findings and those of other studies may respond to the source of the data. Our data were collected for administrative purposes rather than for the research ones and, therefore, it was not possible to evaluate some important variables reported by other researchers.

The main strength of our study was the use of incident cases and patients’ records with a wide variety of socio-economic characteristics, biological and clinical conditions from different health care institutions in charge of tuberculosis control programs, and residence in different communes of the city.

A major limitation was the deficiencies in the quality of the clinical records and their underreporting, which is a situation that did not allow for the collection of some variables reported as important by other studies. Another limitation was that having a secondary information source, studies usually have a bias of non-differential misclassification, which is preferable over a differential misclassification bias toward the null hypothesis. Finally, the lack of genetic tools to differentiate reinfection and reactivation as the cause of relapse is an important limitation.

We believe that the selection bias is unlikely because all cases should be reported to the *Secretaría de Salud Municipal* database; otherwise, patients would be left without treatment. On the contrary, there are two ways of controlling potential confounder variables during the study design and analysis which we tried. Multivariate analysis is the strongest way to control them since the independent variables are usually associated with one another and may have different distributions within levels of the outcome variable. One goal of such analysis is to statistically adjust the estimated effects of each variable in the model for differences in the distribution and associations among independent variables. By applying this concept to a multivariate logistic regression model, it is surmised that each coefficient provides an estimate of the odds adjusting for all other variables included in the model. Nevertheless, there will always be a confounding residual due to variables not included in the model.

In conclusion, we found that BMI and population density were the major risk factors inversely associated with pulmonary tuberculosis relapses. No significant association was found with biological and programmatic factors. To obtain some more evidence in this respect, it is necessary to conduct new studies that include other factors and larger sample sizes.

Based on our findings, we recommend the following to reduce the incidence of pulmonary tuberculosis relapses in Cali:

1) The municipal tuberculosis control program should prioritize strategies to lower BMI;

2) comprehensive health care should be provided while considering the social determinants of tuberculosis, and

3) the tuberculosis control program program should be articulated with other social programs, such as mental health, food security, and nutrition programs.

Eventually, our results could be extrapolated to other places provided that factors such as extreme alcohol intake, BMI, and population density are considered in different settings.

The fact that population density appeared to be a protective factor was an unexpected result requiring further evaluation.
